# Measuring Use of Health-Related Support on the Internet: Development of the Health Online Support Questionnaire (HOSQ)

**DOI:** 10.2196/jmir.4425

**Published:** 2015-11-20

**Authors:** Susanne Mattsson, Erik Martin Gustaf Olsson, Sven Alfonsson, Birgitta Johansson, Maria Carlsson

**Affiliations:** ^1^ Lifestyle and Rehabilitation in Long Term Illness Department of Public Health and Caring Sciences Uppsala University Uppsala Sweden; ^2^ Clinical Psychology in Healthcare Department of Public Health and Caring Sciences Uppsala University Uppsala Sweden; ^3^ Experimental and Clinical Oncology, Department of Immunology, Genetics and Pathology Uppsala University Uppsala Sweden; ^4^ Clinical Psychology in Healthcare, Department of Public Health and Caring Sciences Uppsala University Uppsala Sweden

**Keywords:** social support, questionnaires, oncology, Internet

## Abstract

**Background:**

Social support plays an important role for the perceived health in people with health problems and chronic diseases. Provision of different kinds of support during the disease trajectory is crucial for many people. Online support is ubiquitous and represents a promising modality for people with chronic diseases. There are no existing instruments that measure various aspects of online support.

**Objective:**

The objective of this study was to create a generic questionnaire regarding health-related support online that can be applied to people with various health problems and illnesses. Additionally, we wanted to test the questionnaire in a cancer population to assess its adequacy in the context of severe disease.

**Methods:**

Initial items for the Health Online Support Questionnaire (HOSQ) were inspired by sociologist James House regarding social support. An exploratory factor analysis was conducted in healthy persons or with minor health problems (n=243) on 31 initial items. The scale was reduced to 18 items and the internal consistency and reliability of the scale was examined along with content validity. Further validation was conducted by a confirmatory analysis on the 18-item scale in a cancer population (n=215). In addition, data on demographics, health problems experienced, and Internet use were collected.

**Results:**

The exploratory factor analysis on the final 18-item scale resulted in 2 factors. After scrutinizing the content, these factors were labeled “reading” and “interacting” and they demonstrated good internal consistency (Cronbach alphas .88 and .77, respectively). The factors were confirmed in the cancer population. The response pattern revealed expected differences both between the interaction and reading scales and according to age, gender, education, and health problems thereby supporting the validity of the HOSQ.

**Conclusions:**

The HOSQ may be a reliable and valid instrument for measuring the use of online support for people with health problems, but the results ought to be replicated in more studies to confirm the results for different diagnoses. If the results of this study are corroborated by future studies, the HOSQ may be used as a basis for the development of different forms of support on the Internet.

## Introduction

During the past 2 decades, the use of the Internet as a tool for health-related support has increased [1]. People turn to the Internet for health-related informational support as well as other types of support, both to find out about health problems and to share in other people’s experiences in a similar situation [2,3]. For persons faced with illness, Internet-delivered interventions can be efficient in providing information, appraisal, and emotional support, thus alleviating psychological distress [4-7]. It is important to investigate the diverse needs and expectations of patients to develop such Internet interventions.

### Social Support

Since the mid-1970s, there has been an increasing interest in the role of social support as a coping resource during stressful life events, generating extensive research [8]. The concept of social support has been analyzed in different models and widely elaborated in studies showing that aspects of social support influence health-related quality of life, stress symptoms, and health [8-10]. A theory developed by James House [11] states that social support can be divided into 4 broad classes or types of supportive behaviors or acts: informational, instrumental, emotional, and appraisal. Informational support is advice or counseling that is helpful for coping with personal and environmental problems. Instrumental support takes the form of practical help or economic help. “Appraisal” means information that individuals use in evaluating themselves, such as feedback on performance or information that facilitates social comparison, etc. Emotional support involves empathy, caring, love, and trust. In an online context, the overall label “social support” could be questioned, but the different classes of support described by House could most likely also be found on the Internet [12-14].

### Health-Related Support Online

Health-related support online differs from face-to-face support. Online support can offer anonymity and greater flexibility regarding time and place. However, the support offered face-to-face involves contact with another human being which could imply experiences with other benefits [15]. Factors associated with an increased use of the Internet for health purposes are youth, female sex, higher education, white collar work, visits to a general practitioner during the past year, and long-term illness or disability [1,16]. Being faced with a health problem can be a challenging situation associated with different levels of psychological distress [17] and research suggests that people with chronic disease can benefit from using the Internet for health purposes [18,19].

An increasing trend in health-related Internet use among cancer survivors has been identified [20]. In a recent study in which an online questionnaire with open-ended questions was used describing why cancer patients choose the Internet as a source of social support, it was found that the incentives were, among others, the need for informational and emotional support, lack of support outside the Internet, and the ease of online communication [14].

### Social Support Measures

In an offline context, measures of social support have been developed and applied over the past few decades [21]. Regardless of the underlying model, they typically fall into structural measures, including the network size, frequency or density, and functional measures including emotional, appraisal, informational, and instrumental support. The general finding that stronger social support networks are beneficial has remained relatively constant [21].

Because the use of eHealth services is growing, there is a need to improve our knowledge about how these services are used, by whom they are preferred, and for what reasons. With that information, adequate interventions can be developed and implemented by health providers. To date, however, no questionnaire exists that captures the previously mentioned aspects of support in an online context. Therefore, the development of such a questionnaire is important because the Internet has become such a potentially significant source of health-related support [1,13,22-24]. There is a need for more studies in various contexts to further investigate the incentives for using the Internet for support.

### Aim

The overall aim of this study was to create a generic questionnaire regarding health-related support online that can be applied to people with various health problems and illnesses. Additionally, we wanted to test the questionnaire in a cancer population to assess its adequacy in the context of severe disease.

## Methods

### Questionnaire Development

The development of the Health Online Support Questionnaire (HOSQ) was inspired by the theory of social support developed by House [11] in that his categorization of 4 different classes of support—instrumental, informational, emotional, and appraisal—was used as a tool to guide the nature of the questions.

The first step was to go through the literature regarding support online and search for existing questionnaires measuring aspects of seeking online support. Searching the databases PubMed, PsycINFO, and Scopus resulted in many citations regarding the search for informational support, but no citation mentioning any questionnaire capturing various kinds of potential forms of online support. The second step was to do a comprehensive review of websites related to cancer health issues. With the inspiration of all these sources, we began the process of generating a set of questions encompassing aspects of House’s classes of social support applicable to online support. Three of the authors, independently of one another, scrutinized the suggested questions to see if any aspect of potential support was missing or if there was unnecessary overlap. This yielded the preliminary HOSQ consisting of 31 items regarding the purpose of health-related Internet use (see Multimedia Appendix 1). They were all scored on a 6-point Likert scale describing the frequency of use ranging from zero (not relevant/never) to 5 (on a daily basis).

The first draft of the questionnaire was tested with the so-called “think-aloud method” [25]. Three women with a former breast cancer and 5 men with a former prostate cancer answered the questionnaire while speaking out loud about what they were thinking. They were also given the instruction to share their opinions regarding whether they thought the questionnaire was missing something essential related to the topic and whether it contained anything that was irrelevant or that could easily be misinterpreted. Thus, face and content validity was evaluated. Five of these individuals tested the questionnaire initially and a small revision of the questionnaire was made. Thereafter, another 3 individuals tested the revised version of the questionnaire according to the same procedure, whereupon the final revision before the psychometric testing was done.

After finalizing the draft, an exploratory factor analysis (EFA) using principal axis factoring (PAF) was conducted in a sample from a nonclinical population to investigate whether there was a relevant factor structure underlying the item responses.

In the EFA, reduction of items was guided by the response pattern and statistical considerations, as well as the theoretical constructs. The content of every item was inspected to avoid overlap and items not matching the core aspects of the suggested factors. Further, the explained variance of the questionnaire was balanced against its length to make the HOSQ as clinically acceptable and easy to use as possible (see Multimedia Appendix 1).

### Psychometric Testing

#### Sample and Procedure

To reach out to a heterogeneous group of individuals who were healthy or had minor health problems, we tested the HOSQ in a convenience sample that consisted of staff at a factory in a rural area in central Sweden (n*=*176) and staff at Uppsala University (n*=*67). At the factory, we could reach both laborers and clerks; at the university, we could reach a group of individuals with the highest level of education. That way we could compare differences between groups found in previous studies [13,16]. Data were collected from March to May 2014. Paper copies of the HOSQ were handed out in the job mailbox to the sample at the university and outside the canteen at the factory. In total, approximately 500 questionnaires were handed out and 243 (48.6%) were completed. Participants’ median age was 44 (range 20-71) years and there was a preponderance of men (67.9%, 165/243). In all, 66 (27.1%) participants were single and 177 (72.8%) were living in cohabitation. Regarding education, 105 (43.2%) had a university degree and 123 (50.6%) had high school or elementary school education; 5 (2.1%) did not answer the question relating to education. Those who reported no use of the Internet during the last 2 years (n*=*6) were excluded from all analyses.

A confirmatory factor analysis (CFA) was done in a sample of adult patients (age>18 years) with different cancer diagnoses (n=215). They were recruited at an oncology or urology clinic at a hospital in Uppsala, Sweden. The inclusion criteria were that they could read and understand Swedish and that they had completed the initial treatment (surgery and/or chemotherapy and/or radiotherapy) or were undergoing active surveillance, hormone, or other medical treatment. This was so they had gained some perspective on how they had used the Internet after being diagnosed. Exclusion criteria were that it was their first visit at the clinic and that they were participating in an ongoing Internet-based psychosocial intervention at the hospital, U-CARE [26], that could have an impact on the answers. The data were collected from November 2014 to February 2015. Patients were given a paper copy of the HOSQ in the waiting room and could chose to either answer it at the clinic or complete it at home and return it by mail in a prepaid envelope. Approximately 350 questionnaires were handed out and 285 patients answered the questionnaire. Of these, 70 reported that they had not used the Internet and were consequently excluded from the CFA. The age range in the cancer group was 20 to 84 years and the median age was 63 years. There were slightly more men (120/285, 55.8%). A majority were living in cohabitation (181/285, 84.1%); 31 (14.4%) were single and 105 (48.8%) had a university degree.

In addition to the HOSQ, the participants answered some questions about demographic variables, whether they had used the Internet during the last 2 years, and, in the nonclinical population, whether they had any health problems.

Ethical approval was granted by the Regional Ethical Review Board in Uppsala (November 20, 2013; diary number 2013/436).

#### Statistical Analyses

Exploratory factor analysis with PAF was used to investigate the factor structure of the questionnaire in the nonclinical group. To determine the number of factors to extract, we used parallel analysis. Because factors were hypothesized to correlate, oblique promax rotation was used to retain factors. The criteria for retaining an item were (1) a loading >0.30 on either factor and (2) a loading difference >0.15 between the 2 factors. Items with both factor loadings <0.30 were excluded. To confirm the extracted factor structure, a CFA was then conducted on data from the cancer group. The CFA was conducted with structural equation modeling using robust maximum likelihood estimation and the asymptotic covariance matrix. In the measurement model of the CFA, error terms of measurements loading on the same latent variable were allowed to covary. To estimate model fit, the Satorra-Bentler scaled chi-square, the root mean square error of approximation (RMSEA), the nonnormed fit index (NNFI), and the comparative fit index (CFI) were used. Values for RMSEA <0.08 and NNFI and CFI values >0.95 indicated acceptable model fit [27,28].

The response patterns in relation to the demographic variables were analyzed with the Mann-Whitney *U* test and Spearman rho because the distributions were positively skewed. This was done on the HOSQ total score with a maximal possible score (range 0-90) and subscales (range 0-45) calculated.

## Results

Two of the 31 items yielded a very narrow response range, >90% of the responses being zero (not relevant/never). These items were removed from further analysis. Correlations between the remaining 29 items ranged from ρ=.01 to ρ=.65 and the lowest eigenvalue in a preliminary analysis was 0.174; therefore, the risk of singularity or multicollinearity was considered low.

After an EFA using PAF of the remaining 29 items, results indicated that 2 items should be removed based on the exclusion criteria: (1) a loading <0.30 on either factor and (2) a loading difference <0.15 between the 2 factors. Five items with a difference <0.30 between the factor loadings were removed to further trim the instrument. Another 2 items were excluded after content analysis because they were considered deviant from the core content of the factors. Finally, 2 items considered superfluous were removed to make the 2 subindexes equal in length. The final factor loadings after promax rotation for the remaining 18 items, accounting for 45.3% of the variance, can be seen in Table 1. The Kaiser-Meyer-Olkin measure of 0.85 confirmed sample adequacy. Bartlett’s test for sphericity was significant (χ^2^_153_=1582; *P*<.001), indicating that the interitem correlations were adequate.

**Table 1:** Factor loadings of the exploratory factor analysis (EFA) of the final 18-item version of the Health Online Support Questionnaire (HOSQ) in the nonclinical group (n=229).

I do health-related Internet research...	Factor 1: reading	Factor 2: interacting
To search for information that can improve my health	0.616^a^	0.034
To share information about a disease treatment that I’ve been through	–0.083	0.393^a^
To read about other people’s experience of disease/bad health/a treatment	0.502^a^	0.198
To be able to make more well-informed decisions regarding my health	0.720^a^	0.065
To stay in touch with my friends and colleagues when I’m sick or not feeling well	0.078	0.598^a^
To share practical advice and suggestions regarding my health	–0.054	0.685^a^
To search for information that enables me to better understand physicians and other health staff	0.528^a^	0.083
To search for encouragement from others when I’m stricken by disease or not feeling well	–0.070	0.690^a^
To express my opinion regarding health/disease/care	0.043	0.420^a^
To search for information from different sources to enable the best care	0.730^a^	0.085
To search for compassion when I’m not feeling well	0.093	0.445^a^
To get feedback from others who have, or have had, the same health problem as I have	0.137	0.443^a^
To search for scheduled appointments, addresses or phone numbers to health care	0.619^a^	–0.017
To search for information when I feel worried	0.763 ^a^	–0.124
To keep friends and relatives informed on how I feel	0.030	0.507^a^
To get feedback from friends and relatives on how I’m handling my health situation	–0.038	0.586^a^
To search for the latest research regarding my health situation	0.647^a^	–0.045
To find out whether symptoms that I’ve experienced are dangerous or not	0.890^a^	–0.116
% variance	33.9	11.4
Cronbach alpha	.88	.77

^a^ Indicates factor membership.

After scrutinizing the items, the 2 factors were conceptualized and labeled “reading” (9 items) and “interacting” (9 items). Therefore, the final instrument contained 18 items and 2 subscales. The mean value in the nonclinical sample (n=229) was 12.0 (SD 9.1) for the total score, mean 9.1 (SD 6.5) for the reading subscale, and mean 2.8 (SD 3.9) for the interacting subscale (see Table 1).

**Figure 1:**
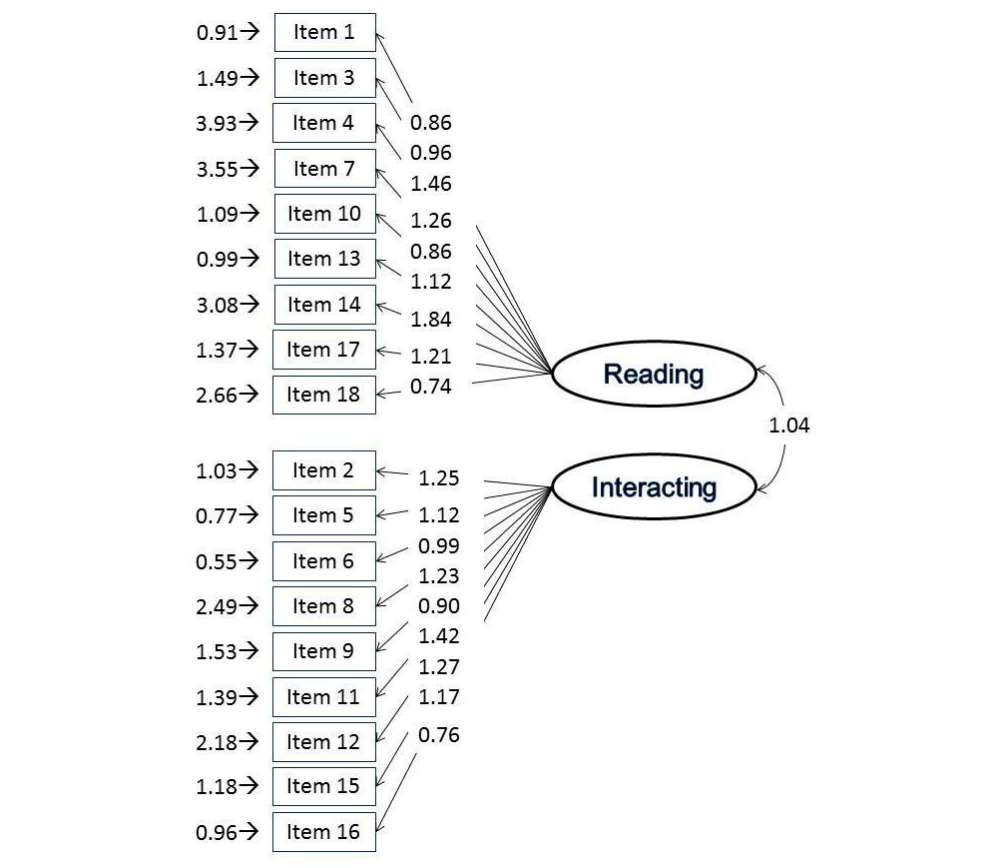
Confirmatory factor analysis of the 18-item Health Online Support Questionnaire with factor loadings, factor covariances, and error variances. Left column: each item of the questionnaire; right column: the two latent variables.

The CFA was conducted with the 18 measurements loading on 2 latent variables, “reading” and “interacting,” and resulted in a significant Satorra-Bentler chi-square (χ^2^_79_=169.6, *P*<.001), RMSEA of 0.073, NNFI of 0.98, and CFI of 0.99 (see Figure 1). Taken together, this indicates an adequate fit for the model and there were no indexes that suggested a potential substantial improvement of the model (see Figure 1).

Women in the nonclinical group scored statistically significantly higher on the HOSQ total score and reading subscale compared to men (Table 2). In the cancer group, women scored significantly higher on the interacting subscale and the HOSQ total score. Participants in the nonclinical sample who reported having a health problem scored significantly higher on the reading subscale and had a significantly higher total score compared with others. Participants with a university education scored higher on the reading subscale in the nonclinical group and on both subscales and the total HOSQ score in the cancer group. There were no differences between single or cohabitating participants. Younger age was correlated with higher scores on the reading and interacting subscales and the total HOSQ score in both the nonclinical and the cancer group (nonclinical group: *R*=–.34 to –.38; cancer group: *R*=–.19 to .27; all *P*<.001).

## Discussion

The HOSQ was found to provide useful information regarding health-related support online and a meaningful 2-factor structure with good internal consistency. The factors represent reading versus interactive eHealth behaviors. This structure was confirmed in a second population with cancer and is, therefore, considered to be robust. The response pattern revealed expected differences both between the interaction and reading scales and according to age, gender, education, and health problems and thereby supports the validity of the HOSQ [22].

### Psychometric Testing

The EFA did not result in the 4 classes suggested by House [11]. A plausible explanation is that the factor structure identified tapping reading and interaction is more salient even though the classes of support suggested by House are embedded in the 2 appearing factors. A majority of the questions measuring instrumental support were removed. This was because of bad fit with the 2 factors derived in the HOSQ. The need for instrumental support should not be overlooked or underestimated in a group of individuals with health alterations or problems, although not central in the HOSQ.

**Table 2:** Medians, third quartiles (Q3), and differences between groups of participants.

Nonclinical population			HOSQ Reading, median (Q3)	*P* ^a^	HOSQ Interacting, median (Q3)	*P* ^a^	Total score, median (Q3)	*P* ^a^
**All (N=229)**			9 (14)		1 (4)		11 (18)	
	**Gender**							
		Women (n=74)	11 (17)	.006	2 (6)	.06	14 (20)	.003
		Men (n=155)	8 (13)		1 (4)		10 (16)	
	**Health problems**							
		No health problem (n=108)	6 (11)	˂.001	1 (4)	.17	8 (16)	.001
		Health problem (n=119)	11 (15)		1 (5)		13 (19)	
	**Marital status**							
		Married/cohabitating (n=168)	8 (13.5)	.28	1 (4)	.41	10.5 (17)	.22
		Single (n=61)	9 (15)		1 (6)		11 (19)	
	**Education**							
		University education (n=100)	10 (15.50)	.02	1 (4)	.98	12 (19)	.06
		≤High school (n=125)	7 (13)		2 (4)		10 (17)	
**Cancer population (n=190)**			8 (17)		2 (6.75)		12 (23)	
	**Gender**							
		Women (n=88)	8 (17.5)	.17	4 (10)	.006	14.5 (26)	.02
		Men (n=102)	7 (14.5)		1 (4)		9 (19)	
	**Marital status**							
		Married/cohabitating (n=160)	8 (16)	.93	2 (6)	.63	12 (22.75)	.89
		Single (n=29)	8 (18)		1 (8)		12 (26)	
	**Education**							
		University education (n=99)	11 (19)	˂.001	3 (10)	.005	16 (28)	˂.001
		≤High school (n=88)	5 (11)		1 (5)		7 (16.5)	

^a^ Mann-Whitney *U* test.

### Health-Related Support

People suffering from a disease primarily search for information, but also visit online networks that offer the opportunity to share in other people’s experiences and to talk about their own experiences. One study examining the exchange of information on Twitter among people with cancer found that it was emotional support rather than information and news that was exchanged [12]. It has been found that those who interact in online networks, rather than just read, report a higher level of mental well-being [29]. Therefore, it is desirable to further investigate the differences between just reading and reading combined with interacting to be able to offer support in accordance with what is searched for.

### Demographic Variables

Women seemed to use the Internet for health-related purposes, especially for interaction, more than men do. Other studies that have shown that women seek health information online more often and have a lower dropout rate in online self-help interventions compared with men [30,31]. Women are also more willing to go online to figure out a possible diagnosis [22] and use social media and blogging for this purpose [32]. Younger people used the Internet for health-related purposes more often than older people in both groups. This is in-line with other research that has found that Internet use is not as high among older adults. Internet use is increasing in the older age group, internationally and in Sweden [22,32,33], potentially meeting the needs in a health care consumer group.

### Health Problems and Online Support

Participants who reported having a health problem searched for health-related information and interaction to a greater extent compared with participants who did not report a health problem. In her theory of online support, LaCoursiere [34] elaborates on the incentives for searching online support. She claims that the search process begins with initiating events, such as an alteration in health status and an alteration in perceived health. Our results for the nonclinical group support her theory. On the other hand, another study found that those who reported poor health used the Internet less for health purposes compared with those who did not [1]. Maybe this has to do with cultural differences in the way that the threshold for health-related Internet use is higher in countries where the access to Internet is relatively low and is correlated to a higher socioeconomic status and better health. In Sweden, approximately 90% of the population older than 12 years have access to the Internet at home [32]. And more than 90% of the population are Internet users, which makes Swedes one of the most Internet-using people in the world.

### Reading Versus Interacting

According to a large survey on Internet use and chronic illness, approximately one-third of a group with chronic disease who consumed health information online reported that they read other people’s comments or about others’ medical/health experiences on online newsgroups, blogs, or websites [18]. Of those, only 6% reported that they posted health-related comments themselves. In that study, the ones who shared knowledge and experiences and interacted with others were a minority compared with the ones who read and took part in what other people shared [18]. People who share information report more benefits from online social support groups and a higher mental well-being than those who do not share [29,35]. The HOSQ may be used in future studies to find out whether individuals or groups tend to be primarily interested in reading only or also to interact. It may also be used together with other instruments to investigate how these different behaviors correlate with other variables. That information might increase the opportunities to offer adequate support online.

### Strengths and Limitations

The confirmatory analysis showed adequate model fit and there was no indication that the model could be further improved. Because CFA can only be used to compare different models, it is important to note that the results of the CFA should be interpreted with caution. Moreover, the sample size used in the CFA was at the lower end of the suggested range, but the model was deemed simple enough to allow for analysis. The factor structure should be confirmed in other, and larger, samples in the future.

One of the limitations of this study is that it was difficult to assess the response rate in all participants because the questionnaire was handed out via the mailboxes of the staff employed at the university. This was easier to control among participants working at the factory and in the cancer group. The vast majority of the participants in both these groups answered the questionnaire; hence, the response rate was high. The minor missing data indicate that the questionnaire was fairly uncomplicated to answer.

Another limitation is that this study used convenient samples and caution should be taken in generalizing the findings to the general population. However the questionnaire was tested in 3 groups: initially in a group of persons with no health problems and in a group with minor health problems (ie, they were not on sick leave) and then in a group diagnosed with a severe disease. Almost half of the nonclinical sample reported having health problems. In other words, the questionnaire was administered to 2 groups experiencing health problems. The same 2 factors appeared in the nonclinical group and the cancer group, indicating that the HOSQ may be a valid as a generic questionnaire examining the search for health-related support online in a population experiencing health problems.

There was a majority of men in both groups. Most of them were well-educated in the nonclinical group, whereas among the women there was a preponderance of well-educated individuals in both groups. This is not fully representative of the overall online population and may have led to higher scores. Women and men with high education are more frequent Internet users [22]; hence, this may have affected the outcome.

Further, we could have used focus group or individual interviews in the initial development process of the questionnaire. Early involvement of key informants is regarded as an important part of questionnaire development and lack of interviews could have influenced the selection of questions. However, the think-aloud method involving patient experts did not indicate a lack of important aspects. Hence, it is reasonable to assume that the HOSQ may have fairly good content validity, but this needs to be more thoroughly tested in future studies.

Construct validity needs to be further evaluated. Having health problems, younger age, and female gender were expected to predict higher total HOSQ scores. This is in-line with other studies measuring searches of health-related information on the Internet. Because the HOSQ is a questionnaire also measuring other types of support, the “known group validity” is not completely applicable. Therefore, there is uncertainty about its construct validity at this point.

Regarding reliability, one limitation is that the questionnaire was administered only once in both the nonclinical and the cancer group. On the other hand, the HOSQ measures behavior. Had we administered the questionnaire twice, there is a risk that the answers could have changed due to altered behavior between the 2 observation points, which would have made it difficult to assess test-retest reliability. Therefore, it is crucial to repeat the testing of the questionnaire in other groups.

### Conclusion

Because of the similar factor structure and salient Internet behaviors in both the nonclinical and the cancer group in this study, the HOSQ may be a promising first step in the development of a generic questionnaire for individuals in various groups with health alterations. However, further tests of the HOSQ’s validity and reliability in other groups are needed to strengthen the presented results. Many people today use the Internet as a tool to help themselves (and others) better understand and handle what is ailing them. Although the Internet is a potentially productive source of health-related support, it is not certain that everyone can benefit from using it. The HOSQ may be used in future research regarding purposes of Internet use and studies regarding reading versus interacting. With the help of that information, tailored support can be developed for different groups or individuals.

## Appendix

Multimedia Appendix 1 Item reduction overview.

## Abbreviations

CFA: confirmatory factor analysis
CFI: comparative fit index
EFA: exploratory factor analysis
HOSQ: Health Online Support Questionnaire
PAF: principal axis factoring
RMSEA: root mean square error of approximation
