# Supplementary material for: Measuring Use of Health-Related Support on the Internet: Development of the Health Online Support Questionnaire (HOSQ)
Source: J Med Internet Res. 2015 Nov 20;17(11):e266. doi: 10.2196/jmir.4425 (PMC4704881; doi:10.2196/jmir.4425)
Supplement: Multimedia Appendix 1 [file jmir_v17i11e266_app1.pdf]

Multimedia Appendix 1. A schedule on the incentives for removing and maintaining the initial 31 items. The items with a bold font are the maintaining items.

Items	Restricted response width	Factor loadings $< .30$ or $\Delta < .15$)	Factor loadings $\Delta < .30$	Content mismatch	Overlap and equalization
**1. To search for information that can improve my overall health**					
2. To feel that I'm not the only one with this type of health situation					X
3. To search for information about psychological support			X		
4. To feel that there is someone who is interested in my thoughts and feelings			X		
5. To search for information about how to change unhealthy behaviour					X
**6. To talk about a treatment for an illness/health condition that I've been through**					
7. To buy medicine, vitamins, supplements, alternative medicines, etc.		X			
8. To express my thoughts and feelings anonymously	X				
**9. To read about other people's experience of a particular illness/health condition or treatment**					
10. To order books about my illness/health condition				X	
**11. To be able to make more informed decisions regarding my illness/health condition**					
**12. To stay in touch with friends and colleagues when I'm sick or not feeling well**					
**13. To share practical advice and suggestions about illness/health**					
14. To handle administrative matters relating to my illness/health (e.g., sick leave)			X		
15. To order equipment, assistance, medical travel, etc.	X				
**16. To search for information so I can better understand physicians and other healthcare personnel**					
**17. To seek encouragement from others when I'm ill or in poor health**					
**18. To express my opinion regarding health/illness/care**					
**19. To search for information from various sources so I can get the best possible healthcare**					
**20. To look for compassion when I'm not feeling well**					
21. To meet others in a similar health situation		X			
**22. To get feedback from people who have or have had the same health problem as I have**					
**23. To search for scheduled appointments, addresses or phone numbers to healthcare providers**					
24. To seek confirmation from others that my health-related decisions are good			X		
**25. To seek further information when I feel worried**					

**26. To keep friends and relatives informed about how I'm feeling**		
**27. To get feedback from friends and relatives on how I'm handling my illness/health situation**		
**28. To search for the very latest research regarding my health situation**		
29. To seek financial support on account of my illness/health condition/care (medical insurance, benefits, funds, etc.)		X
30. To obtain personal advice as to how I should handle my health situation	X	
**31. To find out whether symptoms I have discovered are dangerous or not**		

Note. Bold font indicates factor membership in the final 18-item version. X indicates that the item has been removed due to the criteria in the heading of the column.
